# The HLA-E–NKG2A axis in breast cancer: an emerging NK-cell checkpoint and therapeutic target

**DOI:** 10.3389/fimmu.2026.2001812

**Published:** 2026-10-28

**Authors:** Lvzhu Li, Xiaoxu Han, Lu Lu, Kuan Liu, Yongle Li, Lei Su

**Affiliations:** 1 Department of Breast Surgery, Affiliated Hospital of Hebei University, Baoding, Hebei, China; 2 Department of Anesthesiology, Affiliated Hospital of Hebei University, Baoding, Hebei, China; 3 Department of Radiation Oncology, Affiliated Hospital of Hebei University, Baoding, Hebei, China; 4 Department of Integrated Traditional Chinese and Western Medicine, Affiliated Hospital of Hebei University, Baoding, Hebei, China

**Keywords:** breast cancer, HLA-E, immune checkpoint, immunotherapy, monalizumab, natural killer cells, NKG2A, triple-negative breast cancer

## Abstract

Immune-checkpoint blockade has improved outcomes in triple-negative breast cancer (TNBC), but resistance remains common. Natural killer (NK) cells provide complementary surveillance when classical major histocompatibility complex class I (MHC-I) expression is lost or heterogeneous. HLA-E can inhibit NK cells and subsets of CD8+ T cells through CD94/NKG2A, but can also engage activating CD94/NKG2C. In breast cancer, HLA-E associations depend on classical MHC-I status, while spatial TNBC studies link MHC-I heterogeneity to NKG2A-mediated resistance in experimental models. Evidence varies by subtype and remains predominantly translational; clinical efficacy of NKG2A blockade has not been established, including in the small MIMOSA trial of HER2-positive disease. This Mini Review evaluates breast cancer-specific and cross-tumor evidence, the NKG2A/NKG2C balance, therapeutic combinations, and practical biomarker development. We propose prospective assessment of tumor-cell surface HLA-E, regional MHC-I status, NKG2A-positive effectors, activating-receptor balance, and spatial proximity rather than HLA-E positivity alone.

## 1 Introduction

Immune-checkpoint inhibition has changed the treatment landscape of breast cancer, with the clearest clinical impact in triple-negative breast cancer (TNBC). Pembrolizumab combined with chemotherapy improves pathological and survival outcomes in high-risk early TNBC, while pembrolizumab plus chemotherapy improves survival in selected patients with PD-L1-positive advanced disease (1–4). Nevertheless, responses remain heterogeneous, and PD-L1 expression alone does not capture the cellular, antigen-presentation, and spatial features that determine sensitivity or resistance. Breast cancer immunotherapy therefore requires biomarkers that reflect both adaptive and innate antitumor immunity (5).

Natural killer (NK) cells can eliminate transformed cells without tumor-specific peptide recognition and are especially relevant when classical MHC-I expression is reduced (6–9). NK-cell activity reflects the balance between activating and inhibitory receptors. NKG2A (encoded by KLRC1) forms an inhibitory heterodimer with CD94, recognizes HLA-E, and suppresses NK-cell and selected CD8+ T-cell responses (10–14). Its therapeutic effects consequently depend on the state of both cytotoxic compartments, rather than receptor expression alone.

Breast cancer provides a clinically relevant setting for this checkpoint: altered NK-cell function, NKG2A-enriched populations, context-dependent HLA-E prognosis, and MHC-I heterogeneity have been documented (15–18). More recent breast cancer work suggests tumor-intrinsic influences on predicted HLA-E–NKG2A communication (19). Unlike broader accounts of NK-cell checkpoints, this Mini Review distinguishes breast cancer-specific clinical associations, breast cancer mechanistic studies, and cross-tumor evidence, with emphasis on TNBC; HER2-positive and hormone receptor-positive evidence is less mature. A targeted narrative literature search used PubMed/MEDLINE, publicly accessible scholarly indexing, and reference-list screening for studies available through October 2026 using combinations of ‘breast cancer’, ‘HLA-E’, ‘NKG2A’, ‘NKG2C’, ‘KLRC1’, ‘NK cell’, ‘CD8’, ‘immunotherapy’, and ‘monalizumab’; relevant mechanistic, clinical, and translational studies were prioritized.

## 2 Molecular architecture of the HLA-E–NKG2A checkpoint

HLA-E is a non-classical MHC class Ib molecule whose surface stabilization commonly depends on HLA-I leader-sequence peptides and β2-microglobulin. Foundational studies established peptide-loaded HLA-E as a ligand of inhibitory CD94/NKG2A (10, 11). Upon engagement, NKG2A immunoreceptor tyrosine-based inhibitory motifs recruit SHP-1/SHP-2 phosphatases and attenuate NK-cell degranulation and cytokine release; related inhibition can occur in NKG2A-positive CD8+ T-cell subsets (12–14). **Figure 1** summarizes these receptor and peptide-presentation steps.

**Figure 1:**
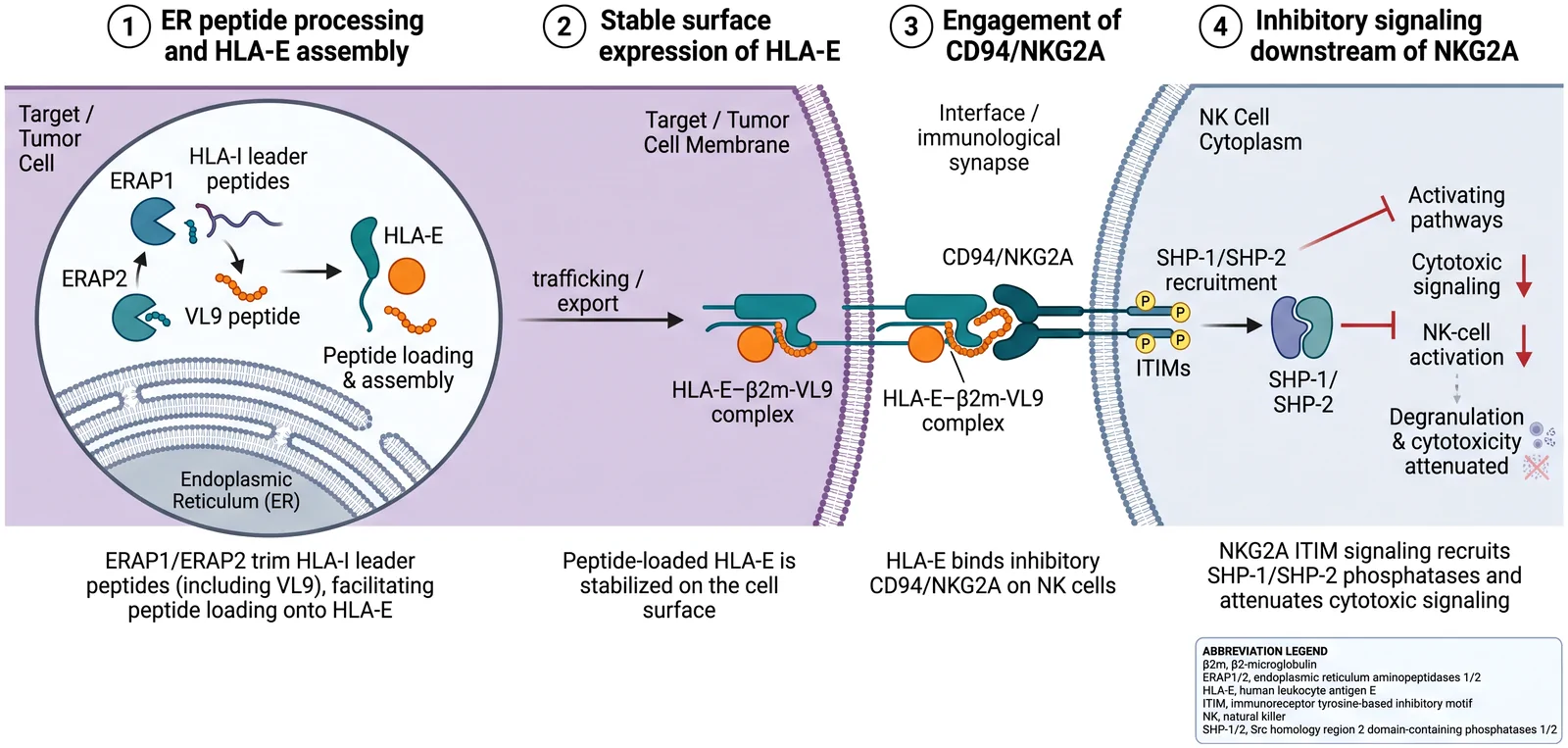
Molecular architecture and functional regulation of the HLA-E–NKG2A checkpoint. (1) In the endoplasmic reticulum (ER), ERAP1 and ERAP2 trim HLA class I leader peptides (such as the VL9 peptide). The trimmed peptides, together with β2-microglobulin (β2m), facilitate the precise loading and assembly of the HLA-E complex. (2) The peptide-loaded HLA-E–β2m–VL9 complex is then trafficked to and stably expressed on the target/tumor cell membrane. (3) At the immunological synapse, surface HLA-E specifically engages the inhibitory receptor CD94/NKG2A on natural killer (NK) cells. (4) This receptor-ligand interaction induces the phosphorylation of immunoreceptor tyrosine-based inhibitory motifs (ITIMs) in the cytoplasmic tail of NKG2A, which subsequently recruits SHP-1 and SHP-2 phosphatases. The activation of these phosphatases blocks intracellular activating pathways, ultimately attenuating NK-cell degranulation and cytotoxic signaling. β2m, β2-microglobulin; ERAP1/2, endoplasmic reticulum aminopeptidases 1/2; HLA-E, human leukocyte antigen E; ITIM, immunoreceptor tyrosine-based inhibitory motif; NK, natural killer; SHP-1/2, Src homology region 2 domain-containing phosphatases 1/2.

HLA-E also binds activating CD94/NKG2C, which signals via the DAP12 adaptor rather than NKG2A inhibitory motifs; receptor affinity, peptide composition, and NK-cell differentiation influence the net response (10–12). NKG2C is enriched in some cytomegalovirus-associated adaptive NK-cell populations with distinctive epigenetic and antibody-dependent effector programs (20). Thus, HLAE RNA abundance cannot establish surface HLA-E function or the direction of its signaling, and NKG2A-positive cells need not be uniformly dysfunctional. Spatial colocalization, protein expression, and the NKG2A:NKG2C balance require separate assessment.

Checkpoint function also depends on antigen processing. HLA-E surface stability requires β2-microglobulin and a suitable peptide repertoire; endoplasmic-reticulum aminopeptidases ERAP1/ERAP2 influence peptide availability. Experimental disruption of this processing altered the HLA-E peptide repertoire and NKG2A-dependent inhibition (21). These results come primarily from broader experimental systems, not breast cancer trials, and therefore provide a mechanistic hypothesis rather than clinical evidence. Total HLAE RNA may remain stable while functional peptide-loaded surface HLA-E changes.

NKG2A can be expressed by activated or tissue-resident CD8+ T cells, and experimental blockade may enhance T-cell as well as NK-cell responses (13, 14). This dual-compartment action motivates combinations with PD-1/PD-L1 blockade but does not establish efficacy in patients with breast cancer. Additionally, the identification of cytotoxic NKG2A-positive γδ T cells at the breast adipose–tumor interface underscores that receptor positivity alone does not identify an inhibitory functional state (22).

## 3 Breast cancer-specific evidence

Early breast cancer studies showed that the prognostic association of HLA-E depends on classical HLA-I status. In a cohort of 677 patients, HLA-E expression was associated with poorer relapse-free outcome mainly when classical HLA-I was lost (15). A subsequent breast cancer immune-phenotyping study found prognostic relevance for invasive ductal, but not invasive lobular, carcinoma, arguing against generalization across histologies (23). These findings concern prognosis, not prediction of response to NKG2A blockade. In a missing-self model, reduced classical MHC-I may weaken CD8+ T-cell recognition while retaining potential HLA-E-mediated inhibition of NK cells.

Breast cancer progression can remodel NK cells. Mamessier and colleagues observed reduced activating receptors (NKp30, NKG2D, DNAM-1, and CD16), increased NKG2A, and impaired cytotoxicity, particularly among tumor-infiltrating NK cells (16). Tumor-draining lymph nodes also contain activated CD16+NKG2A-high NK cells, some co-expressing PD-1 (17). Subtype comparisons have reported distinct NK-cell tolerogenic phenotypes in luminal A, luminal B, and TNBC (24). Recent TNBC single-cell work adds descriptive evidence for heterogeneous immunosuppressive NK states but does not establish therapeutic benefit from receptor blockade (25).

The most direct breast cancer experimental evidence comes from MHC-I-heterogeneous TNBC. Taylor and colleagues linked regional MHC-I heterogeneity to anti-PD-L1 resistance and altered NK:T-cell ratios; dual NKG2A/PD-L1 blockade restored antitumor activity in experimental models and required both NK cells and CD8+ T cells (18). These clinical specimen associations and preclinical intervention results support an emerging model, not a prospectively validated patient-selection rule. MHC-I-low regions may rely more heavily on NK surveillance, which inhibitory HLA-E–NKG2A engagement could constrain.

A 2026 breast cancer study implicated tumor-intrinsic ITGB3-associated states in an HLA-E-rich microenvironment. Its inferred HLA-E–KLRC1/KLRC2 ligand–receptor communication is computational; restoration of NK-derived IFN-γ after NKG2A blockade in co-culture supports functional cytokine modulation but does not, by itself, demonstrate receptor ligation or restored tumor-cell killing (19). Single-cell breast cancer studies further document immune-state heterogeneity or changes during PD-1/PD-L1 therapy (26–29); these should not be interpreted as direct evidence of NKG2A dependence. In radiotherapy-resistant TNBC, an experimental A2AR–phospho-STAT1–HLA-E pathway provides another context-specific hypothesis requiring clinical validation (30).

Evidence is uneven across breast cancer subtypes. TNBC provides the strongest direct association between spatial MHC-I heterogeneity and experimental NKG2A dependence (18). In HER2-positive disease, trastuzumab recruits CD16-mediated antibody-dependent cellular cytotoxicity (ADCC), creating a rationale for relieving NKG2A inhibition; however, MIMOSA reported no objective responses among 11 heavily pretreated patients in its first stage (31). Hormone receptor-positive disease has considerably less pathway-directed evidence, notwithstanding subtype-dependent NK phenotypes (24). The receptor balance, treatment backbone, and functional effector supply must therefore be studied separately across subtypes. Key breast cancer and translational evidence is summarized in **Table 1**.

**Table 1:** Key evidence for the HLA-E–NKG2A axis in breast cancer and translational development.

Study	Setting	Key finding	Translational implication	Main limitation
de Kruijf et al. (15)	Early breast cancer	HLA-E had adverse prognostic relevance mainly when classical HLA-I was lost.	Interpret HLA-E with classical MHC-I context.	Pre-immunotherapy cohort.
Mamessier et al. (16)	Breast cancer progression	NKG2A increased as activating NK receptors and cytotoxicity declined.	NKG2A is part of tumor-induced NK dysfunction.	Not a treatment-prediction study.
Frazão et al. (17)	Tumor-draining lymph nodes	Activated CD16+NKG2Ahigh NK cells were enriched; a subset expressed PD-1.	Supports combined innate/adaptive checkpoint concepts.	Lymph-node biology may differ from primary tumor.
Taylor et al. (18)	TNBC; human spatial data and models	MHC-I heterogeneity tracked with ICI resistance; anti-NKG2A + anti-PD-L1 restored experimental responses.	Strong breast-specific rationale for biomarker-selected dual blockade.	Clinical treatment-selection utility remains unvalidated; therapeutic data are preclinical.
Wang et al. (19)	Breast cancer single-cell and functional study	Inferred HLA-E–KLRC1/KLRC2 communication; anti-NKG2A restored NK IFN-γ in co-culture.	Candidate immune-state mechanism for functional testing.	Predicted interactions and cytokine rescue do not prove tumor killing or clinical efficacy.
Geurts et al. (31)	HER2+ metastatic breast cancer	Monalizumab + trastuzumab was tolerable but produced no objective responses in stage I of MIMOSA.	Unselected combination therapy is insufficient.	Small, heavily pretreated cohort.
Kamiya/Gong et al. (36, 37)	Engineered NK cells	NKG2A silencing or KLRC1 deletion enhanced activity against HLA-E+ targets.	Supports checkpoint-resistant adoptive NK-cell strategies.	Preclinical.
Sætersmoen et al. (39)	Engineered receptor platform	NKG2A/C switch receptor converted HLA-E recognition toward activation.	HLA-E can be exploited as an engineered-cell target.	Preclinical and not breast-specific.

## 4 Therapeutic strategies targeting the axis

Monalizumab is a humanized monoclonal antibody targeting the inhibitory CD94/NKG2A receptor. Experimental NKG2A blockade can strengthen NK- and CD8+ T-cell responses and cooperate with PD-1/PD-L1 blockade or ADCC (13, 32). Clinical experience outside breast cancer remains mixed: a study in advanced gynecologic cancers demonstrated receptor saturation and tolerability but limited objective activity (33), while a phase 1/2 study of monalizumab plus durvalumab in advanced solid tumors reported modest clinical activity (34). These cross-tumor findings should not be treated as breast cancer efficacy data.

The phase II MIMOSA study tested monalizumab plus trastuzumab in heavily pretreated HER2-positive metastatic breast cancer, aiming to enhance trastuzumab-dependent NK-cell ADCC. Eleven patients enrolled in the first stage; treatment was tolerable, but no objective responses were observed and recruitment did not continue (31). Its small, single-arm cohort cannot identify why the regimen lacked activity, nor prove that biomarker selection would have changed the outcome. The negative phase III INTERLINK-1 study of monalizumab plus cetuximab in head and neck cancer adds cross-tumor caution but is not evidence of efficacy or inefficacy in breast cancer (35).

Future strategies may test NKG2A blockade with PD-1/PD-L1 inhibition in biologically selected TNBC, although the dual-blockade efficacy evidence remains preclinical (18). Engineered-cell approaches could also bypass inhibition: NKG2A silencing or KLRC1 deletion enhanced human NK-cell activity in experimental studies (36, 37). In an experimental CD33-directed CAR-NK platform, CRISPR-Cas9 disruption of NKG2A improved antitumor activity; this is CAR-NK, not CAR-T, and was not a breast cancer study (38). NKG2A/NKG2C switch receptors can instead convert HLA-E recognition into an activating signal (39). ERAP-mediated peptide-processing disruption and CBL0137 combinations have also been studied preclinically (21, 40).

For HER2-positive disease, the mechanistic rationale is that trastuzumab engages CD16 on NK cells, whereas HLA-E–NKG2A signaling could counteract that activating input. However, ADCC is modified by NK-cell fitness, inhibitory HLA ligands, Fc receptor state, and the surrounding cytokine milieu (16, 41, 42). These factors are candidate explanations for variability, not established causes of the MIMOSA result. Prospective studies should measure receptor–ligand engagement and functional effector capacity rather than HER2 status alone.

## 5 Biomarker selection for breast cancer

The central translational challenge is identifying tumors with functional HLA-E–NKG2A dependence rather than HLA-E abundance. Tumor-cell surface HLA-E should be distinguished from HLAE transcripts derived from mixed malignant, immune, and stromal cells. Classical MHC-I and β2-microglobulin should be assessed concurrently, especially in MHC-I-heterogeneous TNBC (15, 18). HLA-E expression associated with relapse is prognostic, whereas predicting additional benefit from NKG2A blockade requires treatment-specific clinical validation (23).

Five linked dimensions merit evaluation: (i) peptide-loaded tumor-cell surface HLA-E; (ii) regional classical MHC-I/β2-microglobulin status; (iii) the abundance and phenotype of NKG2A-positive NK and CD8+ T cells; (iv) inhibitory NKG2A versus activating NKG2C expression; and (v) tumor–effector spatial proximity. Multiplex immunofluorescence, spatial proteomics, and appropriately validated single-cell assays may resolve these factors. Importantly, NKG2A expression on γδ T cells or adaptive NK cells cannot be interpreted as suppression without functional context (20, 22).

Treatment-associated immune changes may alter interferon signaling, peptide-loaded HLA-E, classical MHC-I, and NKG2A/NKG2C expression. Consequently, paired pretreatment and early on-treatment biopsies could inform prospective development. No validated composite cutoff, scoring system, prospective predictive algorithm, or companion diagnostic currently exists for NKG2A-directed therapy in breast cancer. The proposed five-dimension framework is a research hypothesis, not a treatment recommendation.

Sampling and modeling also require care. Core biopsies may miss MHC-I-heterogeneous niches, whereas bulk RNA mixes tumor and nonmalignant compartments. A practical study would standardize multiplex protein assays for HLA-E, HLA-I/β2-microglobulin, NKG2A, NKG2C, CD56, CD8, and CD16, then prospectively compare composite versus single-marker discrimination in biomarker-stratified trials. Breast cancer multi-omics studies have proposed computational immunotherapy prediction models, but such models require external validation and are not evidence of NKG2A-specific predictive performance (43). Clinical prediction must show a treatment-by-biomarker interaction; prognostic discrimination alone is insufficient.

## 6 Discussion and future directions

The HLA-E–NKG2A axis is relevant to breast cancer because it may restrain both NK-cell and selected CD8+ T-cell function when classical antigen presentation is heterogeneous (13, 14, 18). Its distinguishing translational question is not whether HLA-E or NKG2A is expressed, but whether their functional interaction limits cytotoxicity in a particular immune niche. General receptor reviews provide mechanistic context (32), whereas disease-specific therapeutic support remains largely preclinical (18).

Major uncertainties include competition between inhibitory CD94/NKG2A and activating CD94/NKG2C, variability in peptide-loaded HLA-E, and the possibility of functionally distinct adaptive NK subsets (20). Indeed, work in melanoma suggests NK cells can, under some immune-excluded conditions, impede checkpoint immunotherapy responses (44). MIMOSA and INTERLINK-1 show that available pharmacologic or clinical evidence has not established efficacy in the studied settings (31, 35). Translational immune phenotyping in other tumors can inform assay design, but not breast cancer treatment response: cervical cancer multi-omics profiling is one example (45).

Disease setting and previous therapy may influence pathway measurements. Early and heavily pretreated metastatic tumors differ in immune context, and primary lesions may not reflect metastatic receptor or ligand expression. Radiotherapy-associated HLA-E regulation has been investigated in TNBC experimental systems (30), but this finding alone does not establish sensitivity to NKG2A inhibitors. Mechanistically distinct tumor-microenvironment resistance programs have likewise been studied in glioblastoma, but their findings must not be transferred to breast cancer as proof of HLA-E dependence (46). Cross-tumor evidence for HLA-E protection of circulating tumor cells is similarly hypothesis-generating (47).

Therapeutic development extends beyond antibodies to genetic KLRC1 deletion, NK-cell receptor silencing, NKG2A/NKG2C switch-receptor engineering, and experimental manipulation of HLA-E peptide processing (21, 36, 37, 39). CAR-NK platforms provide a separate route to augment NK-cell antitumor capacity; these approaches have been reviewed across cancers (48). Breast cancer applicability, on-target functional benefit, and safety of NKG2A modification still require direct testing. The goal is to identify contexts where inhibitory signaling, rather than inadequate effector recruitment or other resistance processes, is rate limiting.

In conclusion, HLA-E–NKG2A is a biologically plausible breast cancer checkpoint with emerging TNBC-specific experimental support but no established clinical efficacy. Its interpretation must include activating NKG2C signaling and distinguish association, functional mechanism, and predictive clinical evidence. A prospectively evaluated panel integrating surface HLA-E, classical MHC-I heterogeneity, NKG2A-positive effectors, NKG2A/NKG2C balance, and spatial proximity offers a testable route toward rational combinations with PD-1/PD-L1 blockade or engineered NK cells.
